# Clinical utility of noninvasive fetal trisomy (NIFTY) test – early experience

**DOI:** 10.3109/14767058.2012.678442

**Published:** 2012-04-28

**Authors:** Tze Kin Lau, Mei Ki Chan, Pui Shan Salome Lo, Hon Yee Connie Chan, Wai Sze Kim Chan, Tik Yee Koo, Hoi Yan Joyce Ng, Ritsuko K. Pooh

**Affiliations:** 1 Fetal Medicine Centre, Paramount Clinic, Hong Kong; 2 BGI-Shenzhen, Shenzhen, China; 3 CRIFM Clinical Research Institute of Fetal Medicine PMC, Osaka, Japan

**Keywords:** Down syndrome, fetal DNA, NIPD, noninvasive prenatal diagnosis, maternal serum

## Abstract

*Objective*: To report the initial experience of noninvasive prenatal diagnosis of fetal Down syndrome (The NIFTY test) in a clinical setting. *Methods*: The NIFTY test was offered as a screening test for fetal Down syndrome to pregnant women with a singleton pregnancy at 12 weeks of gestation or beyond. A satisfaction questionnaire was sent to the first 400 patients. *Results*: During a 6-month period, 567 NIFTY tests were performed. Over 90% of those studied were ethnic Chinese, and the mean age of the women studied was 36 years. The test was performed at 12–13 weeks of gestation in 49.21%. The median reporting time was 9 days. The test was positive for trisomy 21 in eight cases, and for trisomy 18 in 1 case; all were confirmed by fetal karyotyping. There was no false-positive result. Of the questionnaires, 182 completed responses were received. Over 95% had complete or almost complete resolution of anxiety. Except for one, all were satisfied with the NIFTY test, and all indicated that they would recommend the test to their friends. *Conclusion*: The NIFTY test was a highly specific test. Unnecessary invasive tests and associated fetal losses could be avoided in almost all women who have a normal fetus.

## Introduction

Prenatal screening and diagnosis of fetal Down syndrome have become an integral part of obstetric care in many societies. To avoid unnecessary fetal losses associated with invasive prenatal diagnostic procedures, various screening strategies have been developed. Among the most popular one-stage strategies, the first-trimester combined screening provided the best performance with a detection rate of 90% at a 5% false-positive rate [1,2]. Unfortunately, the positive predictive value is still relatively low at about 5%; that is, only about one in 20 to 30 screened positive pregnancies will be confirmed to be truly affected by fetal Down syndrome. The remaining false-positive results induce not only significant maternal anxiety [3] but also “unnecessary” fetal losses due to subsequent invasive diagnostic procedures. Therefore, there was a continuous drive to develop a prenatal diagnostic test without risk of abortion or a screening test with better performance.

Major breakthrough came from the discovery of fetal DNA in maternal plasma during pregnancy [4]. Using latest molecular technology of massively parallel sequencing and power bioinformatics analysis, it is now possible to detect fetal aneuploidies using maternal blood samples with a detection rate for fetal Down syndrome of over 99% at a false-positive rate of less than 1% [5–7]. This high performance has been repeated and reproduced by different research groups in different parts of the world, covering also aneuploidies other than trisomy 21, and in particular, trisomy 18 [8,9]. Although this new test cannot be considered a diagnostic test yet, its performance as a screening test is far better than any of the currently available strategies.

Recently, screening for fetal aneuploidy using this new approach has been introduced into routine clinical practice. The aim of this study is to review the initial experience of clinical application of this test, in particular the acceptance, satisfaction, and logistics.

## Materials and methods

From August 2011, this noninvasive prenatal aneuploidy test, called the NIFTY (noninvasive fetal trisomy) test, was offered to pregnant women as a form of Down syndrome screening test in a private prenatal diagnosis center in Hong Kong. The test was offered to any pregnant women, irrespective of whether they had undergone any other type of Down syndrome screening tests before. Both the information pamphlet and consent form carried an explanation for the benefit of participants that

1. This test was meant to be a screening test for fetal Down syndrome, with a detection rate of > 99% and a false-positive rate of <1%;
2. The risk of trisomy 18 would be assessed and reported as well;
3. The test can be performed anytime afer 12 weeks of gestation;
4. The test was limited to singleton pregnancy;
5. The report would be available within 2 calendar weeks in 90% of cases; and
6. Repeat blood sampling was required in about 3% of cases.

For each patient, a 30-min appointment was given. During the consultation, a detailed counseling was provided. An ultrasound scan was performed to confirm the number of fetuses, fetal viability, fetal size, and major fetal abnormalities. A written informed consent was obtained from all patients. Five ml of maternal peripheral blood was collected into an EDTA bottle. The blood sample was stored at 4°C immediately before further processing. Serum was prepared within 4 h after collection (subsequently extended to 8 h) by a two-step centrifugation protocol. The whole blood sample was first centrifuged at 1600 g for 10 min at 4°C. The supernatant was transferred to sterile 2.0 ml Eppendorf (EP) tubes placed on ice, which was centrifuged again at 16,000 g for 10 min at 4°C. The final supernatant was transferred to new EP tubes, which was temporarily stored in dry ice or store at -20°C before further processing.

All subsequent molecular tests and procedures, including cell-free DNA isolation, library construction, and sequencing, were performed in the clinical laboratory of BGI-Shenzhen, which is ISO/IEC 17025 certified [7]. Each plasma sample was frozen and thawed only once. The report included risk assessment for trisomy 21 and trisomy 18. Specifically, fetal sex was not reported, even on request. Patients were informed of the test report by phone once available.

The clinical details of all subjects who had NIFTY test between August 2011 and January 2012 were reviewed and summarized.

Anonymous postal survey on satisfaction was sent to the initial 400 patients, whose results were available at least 4 weeks before the time of survey. A reminder was sent 2 weeks afer the initial invitation. Invitations were not sent to those patients with a positive NIFTY result.

## Results

During the 6-month period, a total of 567 NIFTY tests were performed. Table I shows the basic patient characteristics. Over 90% of the pregnant women were ethnic Chinese. The maternal age was significantly higher than the normal obstetric population in Hong Kong, with a mean of 36.0 (range: 20–46). Over 67.2% were aged 35 or beyond; 277 cases were primipara. Six had a previous pregnancy affected by trisomy 21, and 10 had a positive family history.

**Table I:** Basic patient characteristics.

Characteristics	Number of cases (%), *N* = 567
Maternal age	
20–24	4 (0.71%)
25–29	38 (6.70%)
30–34	144 (25.40%)
35–39	252 (44.44%)
40–44	127 (22.4%)
≥45	2 (0.35%)
Gestation at NIFTY test	
12W–13W6D	279 (49.21%)
14W–15W6D	122 (21.52%)
16W–20W6D	142 (25.04%)
21 week and above	24 (4.23%)
Previous trisomy 21 pregnancies	6 (1.06%)
Family history of trisomy 21	10 (1.76%)
Ethnicity	
Chinese	524 (92.42%)
Caucasian	29 (5.11%)
Others	14 (2.47%)
Prior Down syndrome screening test	
None	194 (34.22%)
Combined first-trimester NT + biochemistry	288 (50.79%)
First-trimester NT (± other Ultrasound (USG) markers) only	24 (4.23%)
First-trimester biochemistry only	10 (1.76%)
Second-trimester biochemistry only	41 (7.23%)
Other tests, or more than one test	10 (1.76%)
Result of prior screening tests (*n* = 373)	
High risk	179 (47.99%)
Low risk	124 (33.24%)
Result not available at time of NIFTY test	70 (18.77%)

NIFTY, noninvasive fetal trisomy; NT, nuchal translucency.

About half of the NIFTY tests were performed at 12 and 13 weeks of gestation (49.21%). About two third (*n* = 373, 65.8%) of the patients had a prior screening test before the NIFTY test. Interestingly, 70 of these 373 patients (18.8%) attended the NIFTY test even though the report of their prior screening tests were still not available, which implies that NIFTY may well be their first choice test. In 179 patients, the prior screening tests were positive and they reported they would like to use NIFTY in order to avoid the invasive test. On the other hand, 124 patients were still very worried, even though prior screening was negative. Many of them indicated that they would rather have an invasive test if NIFTY test were not available.

There was no significant technical or logistic problem encountered with the implementation of the test. However, since this was a new test, patients indeed had many questions, ranging from scientific basis, technical details, to logistics and reliability. Probably the most important misconception, given the very high accuracy of the NIFTY test, was the belief that they would not need a diagnostic test if the NIFTY test was positive. The pretest counseling on average took 10 min.

The reporting time was within 14 days in 551 (97.18%) subjects, with a mean and median of 9.51 and 9 days, respectively. In 16 cases (2.82%), the reporting time was more than 14 calendar days, including 4 cases (0.7%) in whom a repeat blood sampling was required.

The NIFTY test was positive for trisomy 21 in eight cases, and for trisomy 18 in one case. All except two cases had been screened to be positive by prior test (Table II), three cases by first-trimester combined screening, one by second-trimester dual test, and three by thickened nuchal translucency. In the remaining two, the NIFTY was performed as a primary screening test. Although all patients were fully aware that the NIFTY test has a <1% false-positive rate, most of them challenged the need for karyotyping when they were informed of the positive result. After carefully counseling, all patients agreed for invasive test, and in all the chromosomal abnormality were confirmed. There was no false-positive case. Since most of the NIFTY-negative cases had not yet delivered, we were unable to assess the false-negative rate, which was anyway not the objective of this study.

**Table II:** Characteristics of the nine NIFTY-positive cases.

	Age	Prior screening	NIFTY result	Karyotyping	Outcome
Case 1	40	Dual test (1:19)	High risk T21	T21	TOP
Case 2	35	NT 3.9 mm	High risk T21	T21	TOP
Case 3	36	No	High risk T21	T21	TOP
Case 4	28	FTCS (1:40)	High risk T21	T21	TOP
Case 5	38	FTCS (1:5)	High risk T21	T21	TOP
Case 6	33	NT 4.1 mm	High risk T21	T21	TOP
Case 7	37	FTCS 1:4	High risk T18	T18	TOP
Case 8	36	No	High risk T21	T21	TOP
Case 9	38	NT 3.4 mm	High risk T21	T21	TOP

FTCS, first-trimester-combined screening; NIFTY, noninvasive fetal trisomy; NT, nuchal translucency; TOP, termination of pregnancy; T18, trisomy 18; T21, trisomy 21.

Of the 400 postal invitations, 182 completed responses were received (Table III). Over 95% of the responders indicated that they had complete or almost complete resolution of anxiety over fetal Down syndrome. But there were still three patients who were still having persistent worries although the NIFTY test was negative. Except for one, all patients were satisfied with the NIFTY test, and in particular, 40.7% were very satisfied. All patients indicated that they would recommend the test to their friends and as much as 64.3% of the patients reported they would recommend NIFTY as a primary screening test. As for the reporting time, over 90% considered it to be acceptable, although close to 60% indicated that a shorter reporting time would be better.

**Table III:** Result of patient satisfaction survey.

Survey question	Number (%), *N* = 182
Prior screening test	
No	48 (26.3%)
One screening test	125 (68.7%)
>1 screening test	9 (5.0%)
Primary reason for requesting NIFTY test	
Told to be high or borderline risk, to avoid invasive test	70 (38.46%)
Told to be low risk, still worry	51 (28.02%)
>1 screening tests with conficting result	5 (2.75%)
As primary screening test because it is the best	40 (21.98%)
Just told by her doctor to have the test	16 (8.79%)
How much the NIFTY result helped to reduce her anxiety	
Completely relaxed	95 (52.20%)
Almost completely relaxed. Minimal anxiety which is difficult to quantify.	80 (43.96%)
Helped a lot, but still worry about Down syndrome about once a week	4 (2.20%)
Still constantly worrying about Down syndrome almost everyday	3 (1.65%)
Did not help at all	0 (0%)
Will she recommend NIFTY test to her friends	
Yes, as a primary screening test	117 (64.29%)
Yes, as a secondary screening test	65 (35.71%)
No	0 (0%)
Strength of recommendation	
Very strong	53 (29.12%)
Strong	122 (67.03%)
Weak	7 (3.85%)
Very weak	0 (0%)
Reporting time	
Far too long to be acceptable	4 (2.20%)
Too long, but still acceptable	14 (7.69%)
Pretty acceptable. But shorter would be better	107 (58.79%)
I am OK with the reporting time	57 (31.32%)
Overall satisfaction	
Very satisfied	74 (40.66%)
Satisfied	107 (58.79%)
Neither	1 (0.55%)
Dissatisfied	0 (0%)
Very dissatisfied	0 (0%)

NIFTY, noninvasive fetal trisomy.

## Discussion

Noninvasive prenatal diagnosis of fetal aneuploidy is a long-waited test, and the majority of pregnant women reported hypothetical interest in this test [10]. Even so, as with any new clinical test, the initial introduction in clinical setting might cause significant confusion with unexpected problems. Patient acceptance might not be as good as what we predicted. Therefore, it is important to review the initial experience so that appropriate adjustments can be made early. The objective of this report was to report the early experience in the introduction of this new technology in real clinical setting.

The study subjects were a mixture of three different populations. First are those screened as high risk by other screening tests, such as the second-trimester biochemical test or the first-trimester combined screening test. The major limitation of these tests is the relatively high false-positive rate, about 5%. This category of patients have a strong desire to avoid invasive test. Even with first-trimester combined screening with a detection rate of 90%, only one in 20–30 screened-positive women will carry an affected fetus. Therefore, NIFTY test would help identify these false-positives and avoid unnecessary fetal losses. However, depending on the “markers” making them high risk, their fetus may be at risk of chromosomal abnormalities other than trisomy 21 or aneuploidy. For example, those with a very large nuchal translucency might be at risk of microdeletion syndromes that cannot be identified by the NIFTY test (or in fact by conventional karyotyping as well [11]). Therefore, these patients must be counseled carefully and made to understand the limitations of the NIFTY test.

The second group of subjects were those who have been screened negative by conventional screening tests, which were unable to alleviate their anxiety. Without the NIFTY, many of them in fact indicated that they would have chosen invasive test, which from risk assessment point of view may not be justifed. The very low false-positive rate of NIFTY helped to alleviate their anxiety, without increasing the chance of requiring invasive test. This study confirmed the very high specificity of the NIFTY test. In none of these cases was there a false-positive NIFTY result. We are not suggesting that women who were screened negative by conventional screening test should have undergone a secondary screening, but the availability of NIFTY test certainly helped to avoid many of the unnecessary invasive tests in this group of ultra-anxious patients.

The last group of subjects were those who did not have any prior screening test (including those who attended the free government screening program without waiting for the result). For them, the detection rate and false-positive rates of the NIFTY were significantly better than any other existing screening test. A recent statement issued by the International Society for Prenatal Diagnosis suggested that “before routine MPS-based population screening for fetal Down syndrome is introduced additional trials are needed” [12]. We agreed that the use of this test as a population-based screening program required further evaluation, particularly in terms of its cost-effectiveness. On the other hand, it is beyond doubt that the NIFTY test is significantly better than any other screening tests in use currently, and we should not withhold the access to this test to those women who prefer to use it as the primary screening method. Nonetheless, we must be careful that the positive predictive value, which is dependent on the prevalence of disease, will not be as good as in the high-risk population.

From the above analysis, it is obvious that the NIFTY test is not intended to be a way to get rid of trisomy 21 individuals, but rather a way to minimize the need for invasive test and therefore to avoid unnecessary fetal losses.

Overall, the initial implementation of the test was successful. The patient's satisfaction was overwhelmingly positive, and the majority would recommend the test to their friends. We were able to achieve a test performance better than we claimed, in terms of reporting time, the need for repeat blood sampling, and the false-positive rate.

All of the nine NIFTY-positive cases were confirmed by conventional karyotyping. It is very important for everyone involved in prenatal screening to understand that even if the detection rate and false-positive rate of NIFTY test were 100 and 0.1%, respectively, the positive predictive value would still be only 66.7% in a population with a disease prevalence of 0.5%, (i.e. one of every three positive cases will still have a normal fetus) (Table IV). Therefore, all clinicians and pregnant women must realize that all NIFTY-positive cases have to be confirmed by fetal karyotyping first before any consideration or attempt of pregnancy termination. Although we believe that the false-positive rate of the NIFTY test for trisomy 21 actually is much lower than 0.1% making the positive predictive value even higher, the principle of confirmation by karyotyping must be continued until we have large enough data set to confirm a 0% false-positive rate.

**Table IV:** The chance of an affected fetus given a positive noninvasive fetal trisomy test result, the positive predictive value, is affected by the prevalence of the condition in the test population and the false-positive rate. Whether the detection rate is 99% or 100%, it does not have a significant effect.

PPV		

FPR	Prevalence of 0.2%	Prevalence of 0.5%
1.00%	16.7%	33.4%
0.50%	28.6%	50.1%
0.25%	44.5%	66.8%
0.10%	66.7%	83.4%

FPR, false-positive rate; PPV, positive predictive value.

The major limitation of this study was the small sample size and lack of follow-up of the screened negative cases, making it not possible to assess the false-negative rate. However, this was not the objective of this study. This issue needs to be addressed by a very large clinical study. Nonetheless, most recent studies suggested that the false-positive rate would be below 1%. In addition, these patients were highly motivated to undergo this new test, and therefore their satisfaction may not represent that of the general obstetric population.

In conclusion, the early experience of the noninvasive prenatal diagnosis suggested that this is a test associated with an extremely low false-positive rate, enabling the avoidance of invasive test in virtually all normal pregnancies. Nonetheless, before this test is widely adopted, both the clinicians and pregnant women should be fully aware that a positive test result cannot be considered diagnostic and must be confirmed by karyotyping.
